# Huangqi Decoction reduces liver fibrosis in rats by modulating PI3K/Akt/mTOR signaling and promoting autophagy

**DOI:** 10.17305/bb.2024.11063

**Published:** 2024-09-25

**Authors:** Jingyin Mai, Tianlu Hou, Jiewen Shi, Yang Cheng

**Affiliations:** 1 Emergency Ward, Shanghai Municipal Hospital of Traditional Chinese Medicine, Shanghai University of Traditional Chinese Medicine, Shanghai, China; 2 Institute of Liver Diseases, Shuguang Hospital Affiliated to Shanghai University of Traditional Chinese Medicine, Shanghai, China

**Keywords:** Liver fibrosis, autophagy, Huangqi Decoction, PI3K/Akt/mTOR, apoptosis

## Abstract

Liver fibrosis is a chronic condition caused by various factors, and currently, there are no widely effective treatments. Autophagy plays a crucial role in maintaining liver energy homeostasis, and its disruption can contribute to the development of liver fibrosis. This study investigates the effects and molecular mechanisms of Huangqi Decoction, a traditional Chinese medicine, on autophagy and apoptosis in fibrotic liver tissue. Tissue staining indicated that Huangqi Decoction mitigated CCL4-induced liver injury and apoptosis in rats. Western blot analysis of liver fibrosis markers revealed that Huangqi Decoction significantly reduced the expression levels of alpha-smooth muscle actin (α-SMA), type I collagen, matrix metalloproteinase-2 (MMP-2), and matrix metalloproteinase-9 (MMP-9). Additionally, serum markers of liver fibrosis and biochemical indicators of hepatocyte injury showed that Huangqi Decoction effectively lowered serum levels of hyaluronic acid (HA), lymph node (LN), type I collagen, aspartate aminotransferase (AST), and alanine aminotransferase (ALT). Furthermore, Western blot analysis confirmed that Huangqi Decoction alleviated hepatocyte injury by promoting autophagy and inhibiting the PI3K/Akt/mTOR signaling pathway. In conclusion, Huangqi Decoction enhances autophagy and inhibits apoptosis through the PI3K/Akt/mTOR pathway in rats with liver fibrosis.

## Introduction

Hepatic fibrosis is the scarring of liver tissue following chronic inflammation caused by various liver injury factors [1]. Chronic liver diseases—including cirrhosis, liver cancer, and virus-mediated infections—affect billions of people worldwide. These diseases are usually triggered by fibrosis, primarily through the proliferation of hepatocytes stellate cells (HSCs), and the activation of myofibroblasts, leading to the overexpression of extracellular matrix (ECM) [2]. Activated myofibroblasts and HSCs are the primary cells responsible for fibrotic scar formation. During liver fibrosis, HSCs interact with multiple immune cells that either reside in or migrate to the liver, synthesizing and releasing signaling molecules, such as cytokines and chemokines that regulate liver fibrosis. Developing effective drugs remains an urgent challenge due to the multiple side effects of anti-fibrosis therapies and the difficulty of diagnosing asymptomatic patients.

Autophagy is a classical intracellular energy metabolism and self-renewal mechanism. It serves as an important effector mechanism against pathogen invasion, stress response, and protecting cells from ER stress damage caused by organelle fragments and misfolded proteins. Autophagy plays a key role in biological development and maintaining homeostasis [3, 4]. Disruptions in cellular autophagy are often involved in the pathological processes of many diseases, including neurodegenerative disorders, cancer, and liver diseases [5]. In normal liver tissue, autophagic activity is low, but hepatocyte autophagy is activated during periods of feeding restriction or starvation, providing the necessary energy and nutrients by degrading intracellular misfolded proteins and damaged organelles, thus maintaining cellular homeostasis [6].

Abnormal autophagy is closely associated with the development of liver fibrosis, while moderately activated autophagy offers protection against fibrosis [7]. Although the role of autophagy in liver fibrosis is well-established, current anti-fibrotic treatments have limitations, such as side effects, insufficient efficacy, and failure to target the underlying mechanisms of fibrosis [8]. Therefore, there is a growing demand for alternative therapies that can more effectively target these mechanisms. One promising approach is the use of traditional herbal medicines like Huangqi Decoction, which has shown potential in both anti-inflammatory and anti-fibrotic effects.

Huangqi Decoction originates from the theory of the Renzhai Zhizhi Prescription written by Yang Shiying, a physician during the Northern Song Dynasty [9]. This prescription is based on the principles of supplementing Qi and nourishing Yin, and it consists of seven traditional Chinese medicines: Astragalus membranaceus, Ophiopogon japonicus, Rehmannia glutinosa, Poria cocos, Trichosanthes root, Schisandra chinensis, and roasted licorice. Huangqi Decoction exhibits multiple pharmacological effects [9]. In a previous report, we demonstrated that Huangqi Decoction serum suppressed the proliferation and migration of rat liver sinusoidal endothelial cells by inhibiting angiogenesis through the promotion of Akt/mTOR-dependent autophagy [10]. The present study aims to investigate the effects and molecular mechanisms of Huangqi Decoction on autophagy and apoptosis in liver tissues induced by CCL4-induced liver fibrosis.

## Materials and methods

### Establishment of hepatic fibrosis rat model induced by CCL4

Healthy adult male Sprague-Dawley (SD) rats were acclimatized for one week before the experiment. The rats were randomly divided into five groups (*n* ═ 10): the normal control group (Control), the model group (Model), and the low, medium, and high-dose groups of Huangqi Decoction. Based on previous studies [11, 12] and preliminary experiments, the rats were administered different concentrations of Huangqi Decoction (3.38, 6.75, and 13.5 g/kg).

From the first day of the experiment, the Control group was intraperitoneally injected with 2 mL/kg of olive oil, while the other groups received intraperitoneal injections of 2 mL/kg of 40% CCL4 olive oil solution twice a week (on Tuesday and Friday) for seven weeks. Starting from the 4th week, the Huangqi Decoction groups were administered Huangqi Decoction granules at doses of 13.5, 6.75, and 3.38 g/kg, respectively, while the blank and model groups received distilled water twice a day for four weeks. The study was approved by the Animal Care Committee of Shuguang Hospital, affiliated with Shanghai University of Traditional Chinese Medicine, and followed the guiding principles for the care and use of laboratory animals adopted by the committee.

#### Sample collection

After the final administration, the rats fasted for 12 h, with access to water. After weighing, the rats were anesthetized, their abdominal cavities were opened, and blood was collected from the abdominal aorta. After standing at room temperature for 30 min, the blood samples were centrifuged at 3500 *g* for 10 min at 4 ^∘^C. The serum was collected, stored in cryopreservation tubes, and kept at −20 ^∘^C for later use. The rat livers were extracted and rinsed in ice-cold saline, and the excess water was removed using filter paper. The liver was weighed to calculate the liver index (liver index ═ liver wet weight/body weight × 100%). Two liver tissue samples were taken from the right lobe, fixed in 10% neutral formaldehyde for HE staining and Masson staining, while the remaining liver tissues were quickly frozen in liquid nitrogen and stored at –80 ^∘^C.

### Histology examination

The liver tissues were immersed in 10% buffered formalin for over 24 h, embedded in paraffin, and cut into 5-µm-thick slices. Pathological sections were examined to assess the extent of fibrosis with H&E and Masson’s staining kits. The liver sections were observed under a light microscope (Leica, DM2500, Germany). Representative views of the liver sections were captured. Pathological observations, including H&E staining, Masson’s staining, and Sirius red staining, were subsequently performed. Masson-stained images were randomly captured from ten fields (magnification × 100) in three mice per group.

### Enzyme-linked immunosorbent assay (ELISA)

To investigate the fibrogenic state in CCL4-induced liver fibrosis, the concentrations of aspartate aminotransferase (AST), alanine aminotransferase (ALT), hyaluronic acid (HA), lymph node (LN), and collagen I in the supernatant of liver homogenate were detected using the R&D system ELISA kits, following the manufacturer’s protocols.

### Tunel assay

Liver tissue apoptosis was detected using the TUNEL stain, following the in situ cell death detection kit protocol (Roche, Mannheim, Germany) as per the manufacturer’s instructions.

### Western blot analysis

Equal amounts of protein from cells or liver tissue samples were separated using vertical electrophoresis, then transferred to PVDF membranes (Millipore, USA). The membranes were blocked with 5% non-fat milk in TBS-T (TBS + 0.4% Tween-20) for 2 h. Afterward, the membranes were incubated overnight at 4 ^∘^C with the respective primary antibodies (alpha-smooth muscle actin [α-SMA], collagen I, matrix metalloproteinase-2 [MMP-2], matrix metalloproteinase-9 [MMP-9], Beclin-1, LC3II/LC3I, P62, p-PI3K/PI3K, p-Akt/Akt, and p-mTOR/mTOR) at a dilution of 1:1000, except for GAPDH and β-actin. After three washes with TBS-T, HRP-conjugated secondary antibodies were incubated for 2 h, followed by three more washes with TBS-T for 8 min each. Finally, the blots were incubated with ECL (Proteintech, USA), and band intensity was quantified using Image J software.

### Ethical statement

The experimental protocols were approved by the Ethics Committee of Shanghai University of Traditional Chinese Medicine (approval number: PZSHUTCM2020050216).

### Statistical analysis

All data are presented as mean ± standard deviation (mean ± SD). Differences between groups were analyzed using two-tailed Student’s *t*-tests or one-way analysis of variance (ANOVA). Statistical significance was set at *P < 0.05, **P < 0.01, and ***P < 0.001. GraphPad Prism 8.2 was used for figure generation.

## Results

### Effect of Huangqi Decoction on liver tissue injury in rats with liver fibrosis

**Figure 1.**
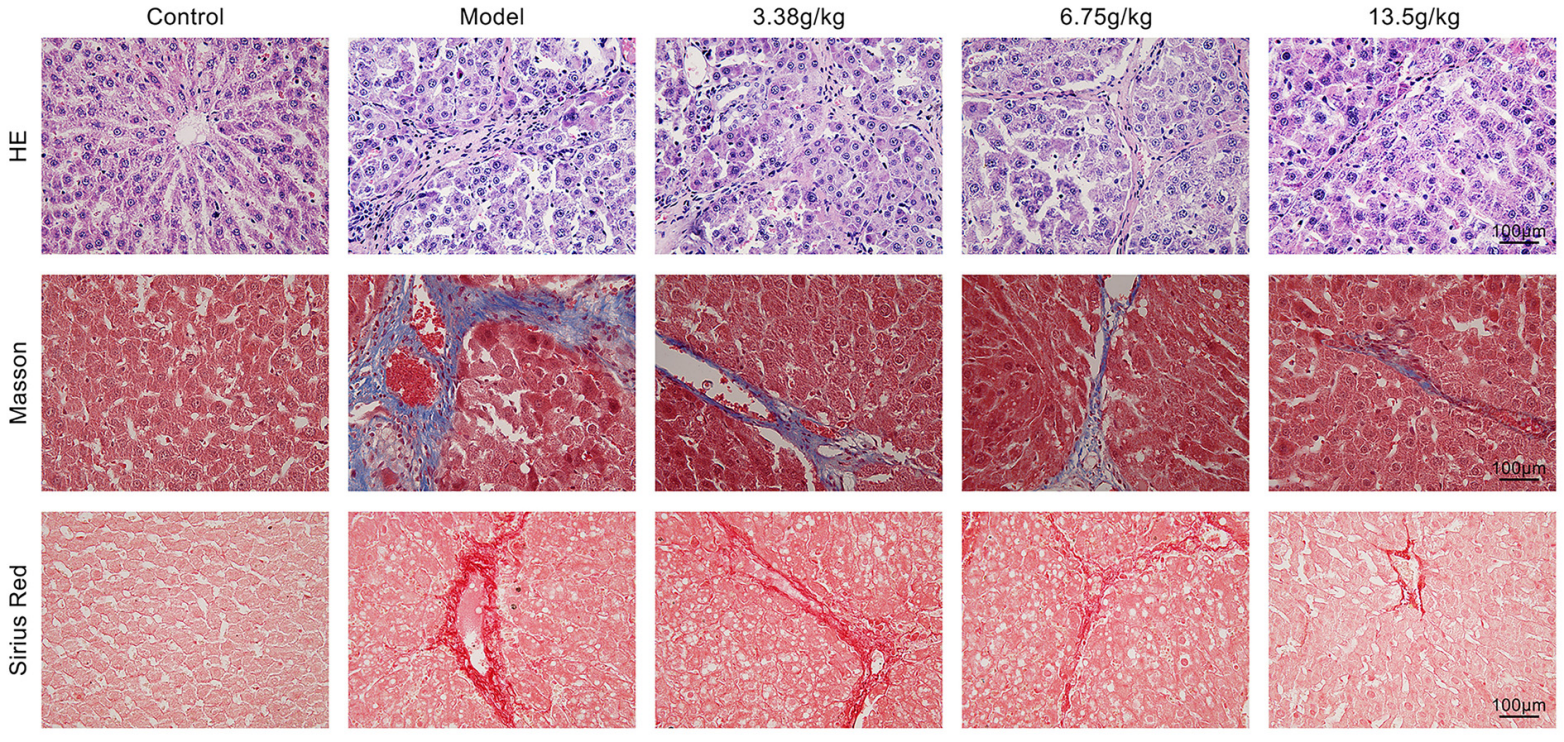
**Effect of Huangqi Decoction on liver tissue injury in rats with liver fibrosis.** The rats were divided into five groups: Control group, Model group, and Huangqi Decoction intervention groups (3.38, 6.75, and 13.5 g/kg). Liver tissues from all five groups were obtained, and HE staining, Masson staining, and Sirius red staining were performed to observe the histological and morphological changes.

To examine the effect of Huangqi Decoction on liver fibrosis, we constructed a hepatic fibrosis rat model induced by CCL4. HE staining was performed to examine the histomorphology of the liver. As shown in Figure 1, hepatocytes were closely arranged with normal morphology and no damage. CCL4 treatment significantly destroyed the liver tissue. However, treatment with Huangqi Decoction at doses of 3.38, 6.75, and 13.5 g/kg effectively improved liver histomorphology. Masson staining and Sirius red staining were also performed. The results showed that, in the blank control group, hepatocytes were neat and regular, the central vein was clearly visible, with a small amount of hepatocyte degeneration, cytoplasmic looseness, scattered necrosis, and mild lymphocyte infiltration in the portal area. In the model group, the liver lobule structure was seriously damaged, hepatocytes were arranged disorderly, significantly denatured, with debris-like necrosis. The portal area was expanded, a large number of inflammatory cells had infiltrated, fibrous tissue hyperplasia was present, and bridging fibrosis in the portal area and around the central vein was observed, along with some fibrous septa and central vein atrophy or disappearance. In the Huangqi Decoction group, hepatocytes were irregularly arranged, with varying degrees of inflammatory cell infiltration, necrosis, edema, degeneration, and fibrous tissue proliferation around the portal area and central vein, but to a lesser extent than in the model group.

### Effect of Huangqi Decoction on liver tissue apoptosis in rats with liver fibrosis

**Figure 2.**
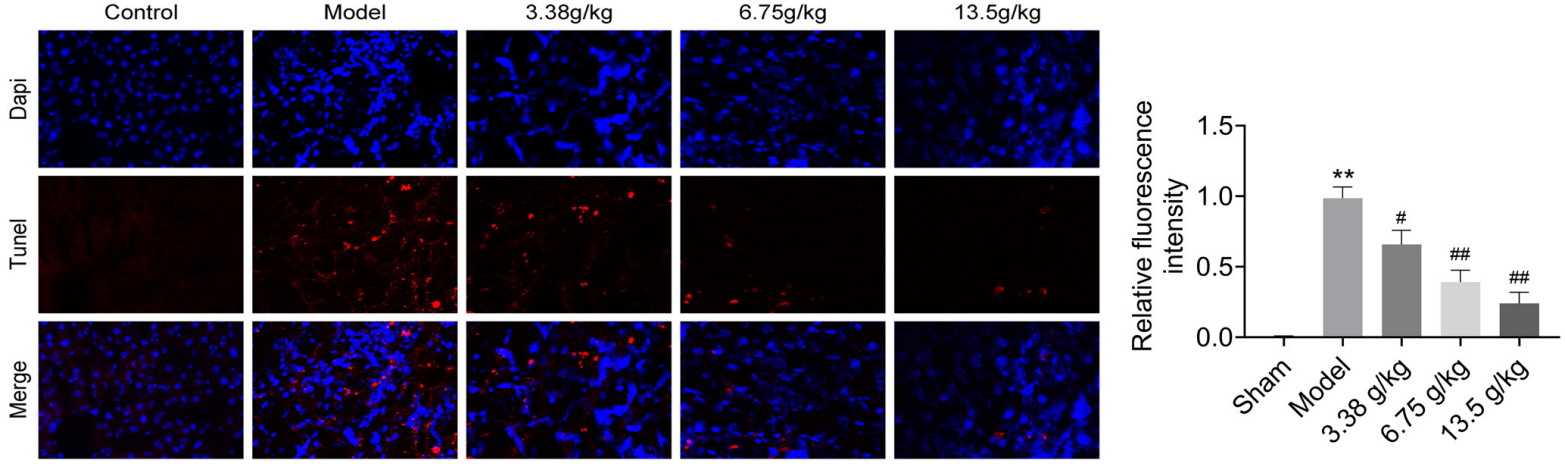
**Effect of Huangqi Decoction on liver tissue apoptosis in rats with liver fibrosis.** Liver tissues from the five groups (Control group, Model group, and Huangqi Decoction intervention groups at 3.38, 6.75, and 13.5 g/kg) were obtained, and the TUNEL assay was performed to observe tissue apoptosis.

We further explored the effect of Huangqi Decoction on apoptosis in liver tissue. Rats were treated with CCL4, followed by Huangqi Decoction treatment (3.38, 6.75, and 13.5 g/kg). Apoptosis in liver tissue was assessed using the TUNEL assay. As shown in Figure 2, compared with the control group, CCL4 treatment significantly induced apoptosis, while Huangqi Decoction intervention effectively reduced apoptosis in a dose-dependent manner.

### Effect of Huangqi Decoction on the expression of hepatic fibrosis markers

**Figure 3.**
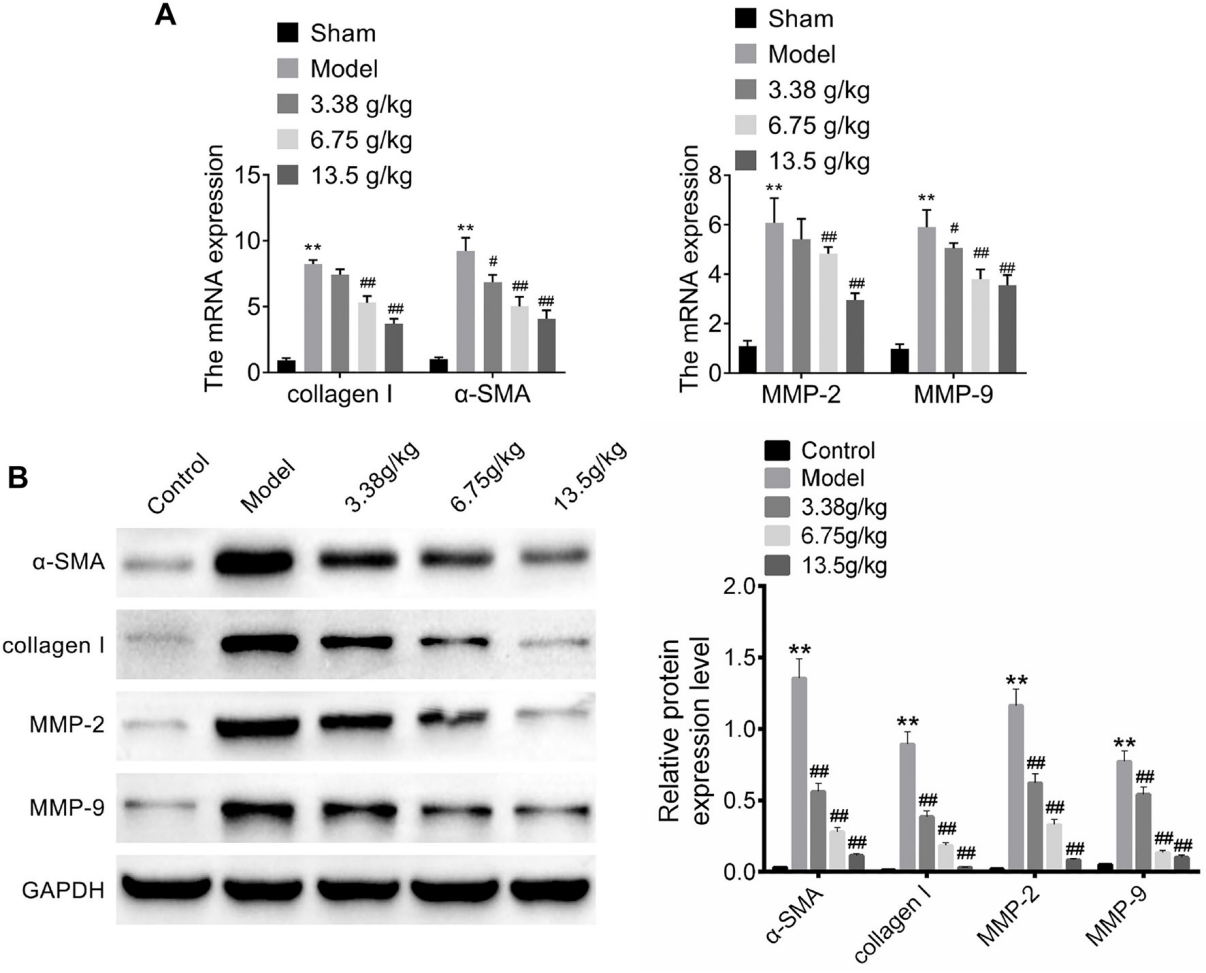
**Effect of Huangqi Decoction on the expression of hepatic fibrosis markers.** (A) The expression of hepatic fibrosis markers (α-SMA, collagen I, MMP-2, and MMP-9) in hepatic tissues from the five groups (Control group, Model group, and Huangqi Decoction intervention groups at 3.38, 6.75, and 13.5 g/kg) was tested by RT-PCR; (B) The expression of hepatic fibrosis markers (α-SMA, collagen I, MMP-2, and MMP-9) in hepatic tissues from the five groups (Control group, Model group, and Huangqi Decoction intervention groups at 3.38, 6.75, and 13.5 g/kg) was examined by western blot. Data are shown as mean ± SD. **P < 0.01 vs Control group; ^##^*P* < 0.01 vs Model group. α-SMA: Alpha-smooth muscle actin; MMP-2: Matrix metalloproteinase-2; MMP-9: Matrix metalloproteinase-9; SD: Standard deviation.

To further evaluate the effect of Huangqi Decoction on hepatic fibrosis in rats, the expression of hepatic fibrosis markers (α-SMA, collagen I, MMP-2, and MMP-9) in liver tissues was examined by western blot [13, 14]. The results showed that CCL4 significantly increased the expression of α-SMA, collagen I, MMP-2, and MMP-9 compared with the control group (*P* < 0.01, Figure 3). However, Huangqi Decoction intervention (3.38, 6.75, and 13.5 g/kg) significantly reduced the expression of α-SMA, collagen I, MMP-2, and MMP-9 in a dose-dependent manner (*P* < 0.01, Figure 3).

### Effect of Huangqi Decoction on protein levels of HA, LN, collagen I, AST, and ALT

**Figure 4.**
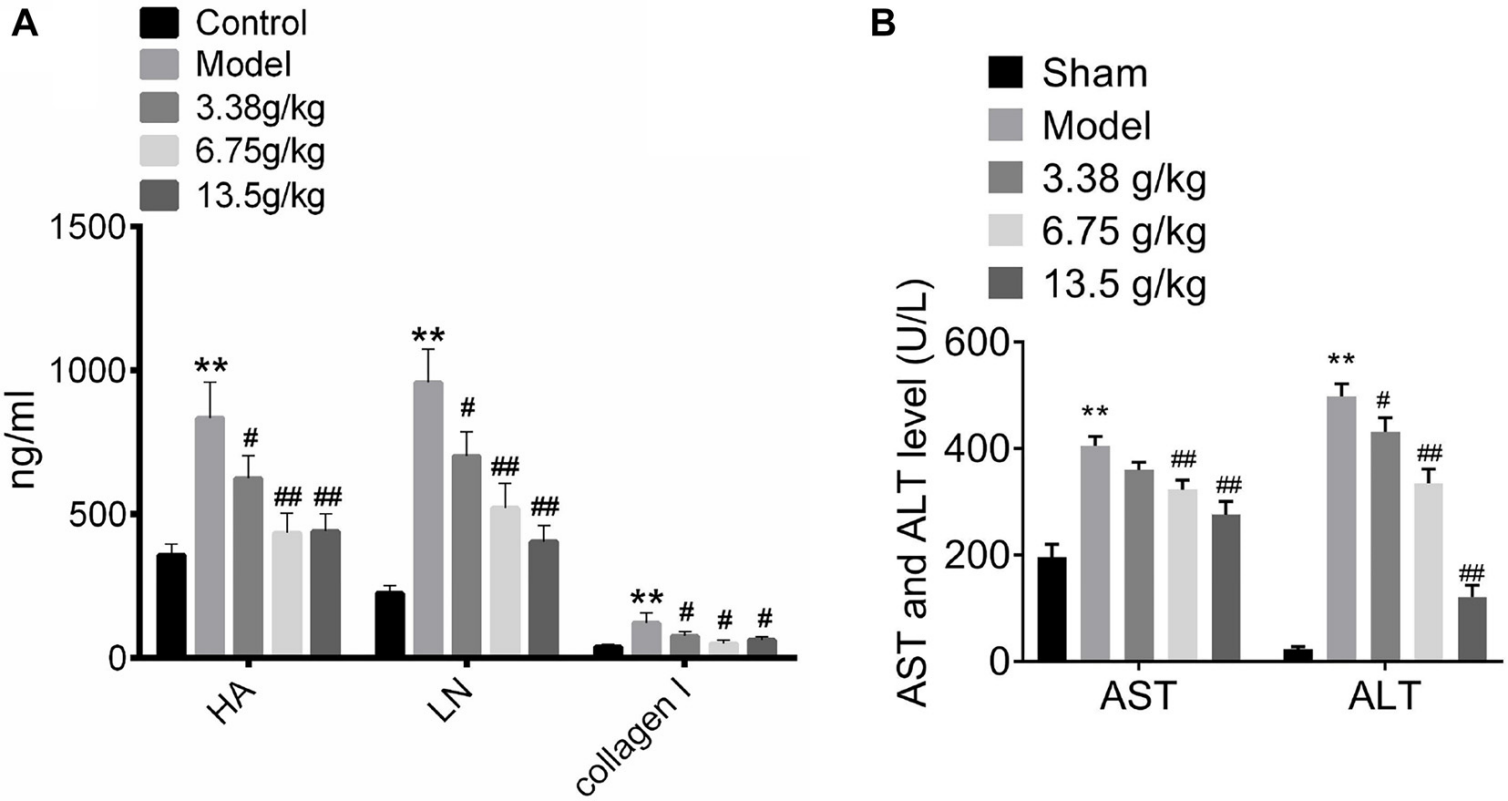
**Effect of Huangqi Decoction on the protein level of HA, LN, collagen I, AST, and ALT.** (A-B) The protein levels of HA, LN, collagen I, AST, and ALT in the blood serum of rats in the Control group, Model group, and Huangqi Decoction intervention groups (3.38, 6.75, and 13.5 g/kg) were detected by ELISA. Data are shown as mean ± SD. **P < 0.01 vs Control group; ^##^*P* < 0.01 vs Model group. HA: Hyaluronic acid; LN: Lymph node; AST: Aspartate aminotransferase; ALT: Alanine aminotransferase; SD: Standard deviation.

We next detected the protein levels of HA, LN, and collagen I in the serum of rats in the control group, model group, and Huangqi Decoction intervention group (3.38, 6.75, and 13.5 g/kg). As shown in Figure 4A, ELISA results indicated that CCL4 induction significantly increased serum levels of HA, LN, and collagen I (*P* < 0.01). However, Huangqi Decoction intervention (3.38, 6.75, and 13.5 g/kg) notably reduced these protein levels to varying degrees (*P* < 0.05). At present, the most widely used biochemical indexes reflecting hepatocyte injury are ALT and AST [15]. The levels of ALT and AST in the serum of rats in the control group, model group, and Huangqi Decoction intervention group (3.38, 6.75, and 13.5 g/kg) were then examined by ELISA assay. The results showed that CCL4 induction significantly elevated the levels of ALT and AST in the serum (Figure 4B, *P* < 0.01), while Huangqi Decoction intervention (3.38, 6.75, and 13.5 g/kg) notably reduced ALT and AST levels to varying degrees (Figure 4B, *P* < 0.05).

### Effect of Huangqi Decoction on autophagy and PI3K/Akt/mTOR signaling

**Figure 5.**
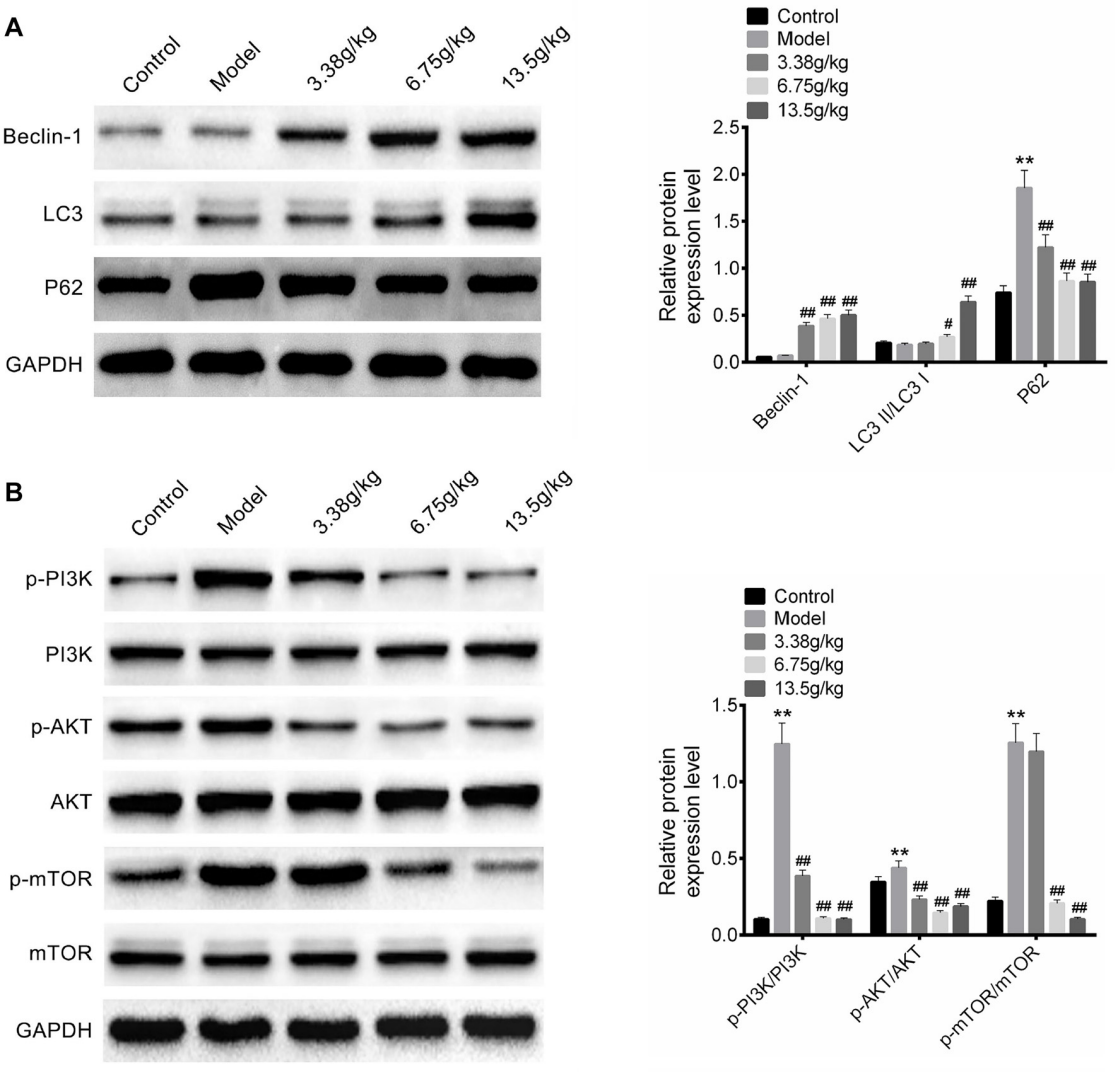
**Effect of Huangqi Decoction on autophagy and PI3K/Akt/mTOR signaling.** (A) The expression of Beclin-1, LC3II/LC3I, and P62 in the blood serum of rats in the Control group, Model group, and Huangqi Decoction intervention groups (3.38, 6.75, and 13.5 g/kg) was detected by western blot; (B) The protein expression of p-PI3K/PI3K, p-Akt/Akt, and p-mTOR/mTOR was detected by western blot. Data are shown as mean ± SD. **P < 0.01 vs Control group; ^##^*P* < 0.01 vs Model group. SD: Standard deviation.

To identify the effect of Huangqi Decoction on autophagy, the expression of Beclin-1, LC3II/LC3I, and P62 was detected by western blot. As shown in Figure 5A, CCL4 treatment had no obvious effect on Beclin-1 and LC3II/LC3I expression compared with the control group. However, compared with the model group, Huangqi Decoction intervention (3.38, 6.75, and 13.5 g/kg) promoted the expression of Beclin-1 and LC3II/LC3I (*P* < 0.05). Moreover, CCL4 treatment increased the protein expression of P62 (*P* < 0.01), while Huangqi Decoction intervention (3.38, 6.75, and 13.5 g/kg) significantly inhibited P62 expression (*P* < 0.01).

The effect of Huangqi Decoction on PI3K/Akt/mTOR signaling was also explored, and the protein expression of p-PI3K/PI3K, p-Akt/Akt, and p-mTOR/mTOR was detected by western blot. As shown in Figure 5B, compared with the control group, the expression of p-PI3K/PI3K, p-Akt/Akt, and p-mTOR/mTOR in the model group was significantly increased (*P* < 0.01). After Huangqi Decoction intervention (3.38, 6.75, and 13.5 g/kg), the expression of p-PI3K/PI3K, p-Akt/Akt, and p-mTOR/mTOR was reduced to varying degrees (*P* < 0.05).

## Discussion

Huangqi Decoction, a traditional Chinese herbal formula, has been widely used for centuries to treat various ailments, including immune disorders, cardiovascular diseases, and liver conditions. Recent studies have provided scientific evidence supporting its efficacy in modulating immune responses and protecting against oxidative stress and inflammation. Previous studies have reported that Huangqi Decoction exhibits various pharmacological actions. Li et al. [16] reported that Huangqi Decoction displayed renoprotective ability by protecting podocytes against apoptosis, regulated through the downregulation of the Nox4/p53/Bax signaling pathway. Wu et al. [17] investigated the effect of Huangqi Decoction on the treatment of alpha-naphthylisothiocyanate (ANIT)-induced intrahepatic cholestasis in mice. They found that Huangqi Decoction protected against intrahepatic cholestasis by reversing the disordered homeostasis of bile acids (BAs) and glutathione. Zou et al. [18] reported that Huangqi Decoction treatment alleviated gut microbiota dysbiosis, ameliorated intestinal barrier dysfunction, inhibited liver inflammation, and protected against DDC-induced cholestatic liver injury. In this study, we explored the effect of Huangqi Decoction on autophagy and apoptosis in liver tissue of CCL4-induced hepatic fibrosis rats. CCL4 is a well-established hepatotoxin that, when administered to experimental animals, induces liver injury and subsequently leads to the development of fibrosis [19]. This model is widely employed in preclinical research to study the mechanisms of liver fibrosis, evaluate the efficacy of potential therapeutic agents, and understand disease progression [20].

In the CCL4 model, repeated exposure to CCL4 causes oxidative stress and lipid peroxidation in the liver, leading to hepatocyte damage and necrosis. This damage triggers a cascade of inflammatory and reparative responses, including the activation of HSCs, which are the primary source of ECM components such as collagen. As HSCs become activated and proliferate, they produce excessive amounts of ECM, resulting in the accumulation of fibrotic tissue and the formation of scar tissue in the liver. The CCL4 model provides a valuable tool for researchers to investigate the underlying mechanisms of liver fibrosis and to test novel therapeutic strategies aimed at reversing or halting the progression of this disease. However, it is important to note that the CCL4 model has its limitations and may not fully recapitulate the complexity of human liver fibrosis.

We found that Huangqi Decoction reduced liver injury induced by CCL4 in rats. CCL4 treatment increased the expression of α-SMA, collagen I, MMP-2, and MMP-9, while Huangqi Decoction reduced the expression of these markers. Huangqi Decoction also regulated the levels of HA, LN, and collagen I. The most widely used biochemical indexes reflecting hepatocyte injury are ALT and AST [15]. Our results showed that Huangqi Decoction inhibited the levels of AST and ALT in the blood serum of hepatic fibrosis rats.

The PI3K/Akt/mTOR signaling pathway is a classical signaling pathway that exists in all cell types and plays an important role in the processes of cell growth, proliferation, and apoptosis [21, 22]. The PI3K/Akt signaling pathway is critical for the activation and proliferation of HSCs, and the pathogenesis of liver fibrosis is based on the activation of HSCs, leading to the deposition of ECM. The PI3K/Akt/mTOR signaling pathway participates in the process of liver fibrosis by regulating the proliferation and apoptosis of HSCs and the synthesis and degradation of ECM [23]. Studies have shown that blocking the PI3K/Akt/mTOR signaling pathway can reduce the adhesion and migration abilities of HSCs, inhibit their proliferation, and reduce the synthesis of ECM collagen, thereby affecting the development of liver fibrosis [24, 25]. Therefore, inhibiting the activation and proliferation of HSCs and inducing apoptosis of activated HSCs by targeting the PI3K/Akt/mTOR signaling pathway has become one of the main strategies for treating liver fibrosis. The PI3K/Akt/mTOR pathway regulates cell proliferation, growth, apoptosis, and metabolism by phosphorylating several substrates. PI3K activates Akt, which is phosphorylated to regulate a variety of downstream targets [26]. Our results showed that CCL4 significantly increased the phosphorylation levels of PI3K and Akt in rats, suggesting that the PI3K/Akt/mTOR signaling pathway was activated. However, Huangqi Decoction significantly decreased the phosphorylation levels of PI3K, Akt, and mTOR in rat liver, indicating that Huangqi Decoction plays an anti-hepatic fibrosis role at least in part by inhibiting the PI3K/Akt/mTOR signaling pathway.

Our study demonstrates that Huangqi Decoction exerts its anti-fibrotic effects through the modulation of the PI3K/Akt/mTOR signaling pathway. This finding is significant because it provides a mechanistic basis for the therapeutic potential of Huangqi Decoction in treating hepatic fibrosis. By elucidating the molecular mechanisms involved, our research contributes to the understanding of how natural compounds can be used to target specific pathways implicated in liver diseases. The identification of the PI3K/Akt/mTOR pathway as a key target for the anti-fibrotic effects of Huangqi Decoction represents a novel contribution to the literature. This advances the field by suggesting new therapeutic strategies that can be explored for the management of hepatic fibrosis. Our findings provide a rationale for further investigation into the use of herbal medicines in treating liver diseases, bridging traditional and modern medical approaches. Clinically, our results suggest that targeting the PI3K/Akt/mTOR pathway with natural products like Huangqi Decoction may offer a safe and effective alternative or complementary therapy for patients suffering from hepatic fibrosis. Understanding the mechanism by which Huangqi Decoction reduces fibrosis and apoptosis can inform the development of more targeted and personalized treatment regimens. Future studies could focus on the detailed molecular interactions between the components of Huangqi Decoction and the PI3K/Akt/mTOR pathway to optimize its therapeutic effects. Additional clinical trials are warranted to validate the efficacy and safety of Huangqi Decoction in human subjects, paving the way for its potential integration into clinical practice. While conventional pharmacological agents, such as corticosteroids and immunosuppressants, have shown efficacy in managing symptoms of hepatic fibrosis, they often come with side effects and do not specifically target the underlying pathogenic mechanisms. In contrast, Huangqi Decoction, by modulating the PI3K/Akt/mTOR pathway, offers a targeted approach that addresses fibrogenesis directly. Some antifibrotic drugs currently under investigation, such as pirfenidone and nintedanib [27], have shown promise but are primarily used for pulmonary fibrosis and require further validation for hepatic applications. Huangqi Decoction, with its natural composition and fewer side effects, presents a safer and potentially more versatile option. Unlike synthetic drugs, Huangqi Decoction is derived from natural herbs and has a long history of use in traditional medicine, suggesting a favorable safety profile.

## Conclusion

In conclusion, our study indicates that Huangqi Decoction exerts a hepatoprotective effect against CCL4-induced injury, promotes autophagy, and suppresses apoptosis through the PI3K/Akt/mTOR signaling pathway in hepatic fibrosis rats.

## Data Availability

The data used to support the findings of this study are available from the corresponding author upon request.
